# An Account of the Mode of Performing the Lateral Operation of Lithotomy

**Published:** 1830-01-01

**Authors:** 


### An Account of the Mode of Performing the Lateral Operation of Lithotomy. With Illustrations. By *Edward Stanley*, Assistant Surgeon, and Lecturer on Anatomy and Physiology at Saint Bartholomew's Hospital. Royal 4to. bds. pp. 23. 7 plates. Longman's; London, 1829.

Among the many peculiarities and advantages of the present age of books and steam engines, we know of none that excite more pleasing reflections than the spectacle of men of eminence in the literary world and their respective professions engaged in the compilation of works for the instruction of youth. Who is not delighted at seeing the amiable and illustrious host of Abbotsford, the high minded Sir Walter Scott, lay his border-harp and minstrelsy aside, forget the knightly deeds of Cœur-de-Lion, or the trials of Jeanie Deans, and employ himself in writing Tales for his Grandchildren? Who amongst our readers that have seen the volumes of the Family Library do not envy the young circles for whom it is executed by such men as Lockhart, Allan Cunningham, and Milman? Amongst ourselves the same gratifying prospect is daily presented to our view, and the veriest cynic, or most callous man of the world must surely experience some of the kindlier feelings of human nature, when such men as Sir Astley Cooper and Abernethy are engaged in the task of publishing their Lectures for the benefit of the students of that profession, over which they have themselves thrown so bright a gleam of honour. We were led into this train of thoughts from perusing the Essay of Mr. Stanley, the title of which we have copied. The object of the author is so well explained in his short and modest preface, that we cannot refrain from transcribing it entire.

"Respecting the operation of Lithotomy, Mr. Cheselden observes, 'If I have any reputation in this way, I have earned it dearly; for no one ever endured more anxiety and sickness before an operation; yet, from the time I began to operate, all uneasiness ceased; and if I have had better success than some others, I do not impute it to more knowledge, but to the happiness of a mind that was never ruffled or disconcerted, and a hand that never trembled during any operation.'

"If these were the feelings of Mr. Cheselden, with his knowledge and experience, at each repetition of the operation for the stone, we are not to be surprised at the anxiety which most individuals endure the first time they perform it. In endeavouring to be serviceable on such an occasion, I have experienced the want of a simple, yet sufficiently detailed account of the mode of performing the operation, unencumbered by critical or historical matter. Such an account it has been my object to supply, and with the hope of having increased its utility, I have added some illustrations of the parts concerned in the operation, in their healthy and diseased states."—*Preface.*

Of course such a work, though commendable alike for the spirit in which it is conceived and the manner in which it is executed, is not altogether adapted for analysis in this Journal, as our object has always been to make these pages rather worthy the study of the old and established practitioner, than the mere perusal or amusement of the student. Nevertheless we shall give the more important points connected with the operation of lithotomy, in order to benefit such of our young friends as may not have the means or the opportunity of consulting the work of Mr. Stanley.

### I. OF THE MODE OF PERFORMING THE OPERATION.

Our author lays down very excellent rules on this subject, rules which perhaps may be read with advantage by older surgeons than those for whose use the work is professedly designed. Mr. Stanley recommends that warm water should be freely injected into the rectum three hours before the operation, and that the bladder should contain some urine at the time of its performance, although it is well known that Cheselden entertained a contrary opinion. The bandages should be applied before the staff is introduced, in order that the position of the latter should not be altered; the staff itself should neither be pressed upwards, downwards, nor to one side, but held firmly, perpendicularly, and without any change of position; the assistant holding the staff generally stands upon the patient's right side, but by standing on the opposite, he has the advantage of holding the instrument in his right hand; two other assistants at the sides of the patient should grasp his knees and feet, and another still should be placed at his shoulders; the pelvis being brought to the table's edge the anterior superior spines of the ilia should be maintained throughout in a horizontal line; before making the incisions the operator should ascertain the situation of the tuberosity and ramus of the left ischium, and then recollecting that the corpus spongiosum is directly behind the raphé of the perineum, he should mark with precision the triangular space between the corpus spongiosum and crus penis, through which the incisions are to be directed to the membranous portion of the urethra.

"The first incision is to be commenced an inch and a quarter above the anus, and a little more than a quarter of an inch to the left of the raphé, and is to be continued obliquely, outwards and downwards, to the extent of about three inches, towards the tuberosity of the ischium. The thumb and fore-finger placed against the perineum will mark the points where the incision should begin and terminate.*

"The first incision should be through the skin, subjacent fat, and tendinous fibres. Its length should never be much less than three inches, and in the case of an unusually large stone, it must be extended towards the tuber ischii even to four inches. Its exact line should be one third from the tuberosity and ramus of the ischium, and two thirds from the anus. Any deviation from this had better be by an approximation of the incision to the tuberosity, than to the anus.

"In a thin person, a scalpel may be plunged through the integuments directly into the membranous portion of the urethra; but, with a considerable depth of fat in the perineum, this is difficult, and in attempting it the bulb will be almost certainly divided, its artery wounded, and the urethra penetrated anterior to its membranous portion.

"With the left fore-finger in the wound, the incisions are to be continued to the membranous portion of the urethra. These incisions, being made in the triangular space between the corpus spongiosum and the crus penis, will divide the transversalis perinei muscle, branches of the perineal artery, a small part of the triangular ligament, and the front edge of the levator ani muscle.

---

* "It is necessary to observe, that this, with the other directions for the operation, is designed for the adult."

"The left fore-finger and the scalpel moving together towards the membranous portion of the urethra, and the staff being felt, with the edge of the scalpel turned upwards, a slit in the urethra is then to be made, to the extent of half an inch at least.* The nail of the fore-finger passing by the side of the scalpel into the groove of the staff, is to remain there as a guide to the beak of the knife or gorget, whichever instrument may be used to complete the operation.

"That there may be a certainty of opening the urethra in its membranous portion, let it be recollected, that the scalpel must enter the staff directly under the symphysis pubis: of the importance of this rule there can be no doubt. Mr. Blizard,—the dexterity of whose numerous operations at the London Hospital has been acknowledged in the strongest terms by all who witnessed them,—attributes his success to the rule of invariably penetrating the urethra in its membranous portion. Mr. Martineau, who has recorded the result of his great experience, states: 'I introduce the point of the knife into it (the groove) as low down as I can, and cut the membranous part of the urethra."† The nearer to the prostate gland the urethra is opened the better, as it lessens the risk of wounding the bulb or its artery.

"As soon as the urethra is opened, urine may flow through the wound: this must be recollected; otherwise it might be supposed the bladder had been opened when the incisions have not reached the prostate.

"The knife being conducted along the left fore-finger to the staff, its beak is to be moved a little within the groove, to ascertain that the two instruments are in contact. The staff, then taken in the left hand, is to be held firmly and without any change of its position, whilst, with the right, the knife is urged slowly onwards to the bladder. Its entrance into the bladder will be indicated by the sudden removal of resistance to its progress, and, probably, by a gush of urine. The incision of the prostate is yet to be adequately enlarged in withdrawing the knife from the bladder, and at the same time directing its blade outwards and downwards. This incision through the left side of the prostate will correspond with the incision of the outward parts, and it should be of sufficient extent to permit the easy passage of the finger into the bladder.

"The knife should be held lightly as it is withdrawn from the bladder, that the operator, sensible of the resistance it meets, may judge accurately of its progress through the prostate. In withdrawing the knife, attention should be given to the position of its handle, which may be inclined towards the left ischium, but not towards the right, as the blade would then be directed towards the pudendal artery." 8.

The gorget is to be conducted into the bladder in the same manner as the knife but its width renders any division of the prostate on its *withdrawal* unnecessary. The scalpel used for the first incisions has been used by some operators to divide the prostate, but Mr. Stanley doubts whether even the steadiest hand will conduct it along the groove of the staff with the same precision as an instrument to the end of which a beak is appended. Some surgeons whilst cutting into the bladder depress the handle of the staff in order that by the elevation of its other extremity any risk of injuring the lower part of the bladder may be avoided; for this position of the staff a cor-

* "It is here presumed, that a single-edged scalpel has been used for the first and subsequent incisions. Then, to secure a free incision of the urethra, the edge of the scalpel must be turned upwards; but with a double-edged scalpel this movement would be unnecessary.

† "Med. Chir. Trans. vol. xi."

responding depression of the handle of the knife or gorget will be required to secure the continued adaptation of the two instruments. The staff may be left in the hands of the assistant until the incision of the prostate is completed, by which means the operator can pass his left fore-finger along the side of the knife whilst dividing the prostate, and ascertain if this incision of the gland is sufficient. Mr. Stanley, however recommends the operator to take the staff himself, as is generally done, and we think there can be no doubt of the general propriety of the advice.

"The knife or the gorget having been withdrawn, the staff is to be taken in the right hand, and the left fore-finger passed along it into the bladder. The staff is then to be withdrawn, and it is to be ascertained by the finger to what part of the bladder the forceps should be directed, that they may immediately touch the stone. On withdrawing the finger, the forceps, with their blades closed, are to be passed slowly through the wound, and inclined upwards as they approach the bladder, carefully observing when they enter its cavity.

"Upon the foregoing plan, the staff will be the conductor of the finger into the bladder, and the finger the conductor of the forceps. The introduction of the finger is useful to ascertain the situation of the stone, and, by separating the sides of the incision in the prostate, to facilitate the passage of the forceps. By allowing the staff to remain in the bladder until the finger has entered its cavity, the beaked or probe-pointed knife can be readily conducted to the bladder, for the purpose of enlarging the incision of the prostate, should this have been inadequately made; and if, from an unusual firmness of the prostate, the sides of the incision through it do not readily yield, much difficulty may be experienced in discovering the passage to the bladder, when the staff has been withdrawn, and its aid, as a conductor of the finger, is thereby lost. So many sources of difficulty are avoided by a strict adherence to the rule of not withdrawing the staff until the finger has fairly entered the bladder, that it cannot be pressed too strongly upon the attention of the operator.

"When the blades of the forceps happen to be passed to the upper or to the under surface of the stone, it may be seized directly they are opened. But when the forceps touch the stone only on its anterior surface, it is necessary, in opening the blades, to advance them a little, otherwise, in separating, they will recede from the stone.

"When the first attempt to seize the stone has failed, the forceps should be directly withdrawn, and the finger introduced into the bladder, to change the situation and position of the stone. But the finger may not reach the stone: a scoop or other instrument must then be introduced, to dislodge the stone from its unfavourable situation; and it is scarcely necessary to mention the importance of introducing into the bladder but few instruments, and these as gently as possible. A stone lodged in the lower part of the bladder has in some instances been raised, and its position changed, by the finger introduced into the rectum." 12.

But, as Mr. Stanley justly remarks, rules will avail little to meet the various difficulties which may arise in the progress of this dark operation, and much must depend after all on the tact and dexterity of the operator. The forcible contraction of the bladder on the stone may resist its seizure:—or the stone may be partially encysted or lodged within the orifice of the ureter:—or it may adhere in some degree to the mucous membrane of the bladder:—or there may be an hour-glass contraction of that organ. In most of these cases the peculiarity is discovered during the operation, and some may be generally suspected from the occurrence of any unusual circumstance in sounding the patient.

"Whether the operation has been completed by a knife or by a gorget, some resistance to the progress of the stone through the wound may be expected. This resistance may be in the prostate or in the external parts; but in either case, it will yield to the skilful use of the forceps, provided that the incisions have been properly made, that the stone is not unusually large, and that the prostate has undergone no change in its structure.

"The skilful management of the forceps consists in pressing their blades gently against the sides of the wound, first in one direction, then in another, but especially downwards*, and in drawing them outwards slowly, that time may be allowed for the yielding of the surrounding parts. Laceration, or severe contusion, of the sides of the wound will probably be followed by suppuration, which, by spreading extensively through the surrounding cellular tissue, may be destructive of life. With these circumstances in his mind, the operator must judge to what extent the effort to extract the stone may be safely persevered in, and when he ought to desist, for the purpose of enlarging the wound with the knife in the situation where there is the most resistance.

"When the resistance to the progress of the stone is in the prostate, although the left side of the gland has been divided to the usual extent, it will be better to cut into its right side than to extend the incision of the left at the risk of wounding the pudendal artery, or of cutting the coats of the bladder beyond its neck.†

"When the resistance to the progress of the stone is in the external parts, the incision of these must be enlarged towards the tuberosity of the left ischium.

"The extraction of the stone must be directly followed by the introduction of the finger into the bladder, for the purpose of examining every part of its internal surface; and this must be carefully done, as there may be a second stone not readily discoverable in consequence of its being lodged in a recess of the bladder at its fundus or elsewhere.

"And it is necessary to examine the surface of the stone. A particle of it may have been chipped off, and retained either in the tract of the wound or in the bladder. Unless it can be discovered in the wound, and thence dislodged by the end of the finger, a stream of warm water must be directed with a good syringe through the wound to the cavity of the bladder, to detach the particle of stone which may be sticking to its mucous membrane." 15.

### II. OF THE INCISION OF THE PROSTATE.

The incision of the prostate is the most debateable part of this debateable operation, and we believe that upon it depends the chief risk of failure or chance of success. As, however, the object of the present article is to explain the ordinary and, cæteris paribus, the best method of performing lithotomy, we shall not enter into the litigated questions respecting the extent of the prostatic incision. Mr. Stanley observes that it can scarcely be doubted that the best direction, is the division of its left lobe obliquely outwards and downwards, and that an incision in the prostate cannot be made in any other direction with so little risk of injury to the

* "This will be towards the wider part of the space between the rami of the ischia.

† "The thin coats of the bladder can scarcely resist the extraction of the stone, and from the incision of the bladder beyond its neck, there will be the danger of infiltration of urine into the pelvis, and of injury to the ureter, so close to the neck of the bladder is its termination in some subjects."

pudendal artery or to the rectum. An incision capable of easily admitting the finger into the bladder is generally sufficient, and if the stone is unusually large, Mr. Stanley well observes that it is better to determine on the incision of both sides of the prostate, than prolong too far the incision on the left side. By this means an increase of space will be obtained, to be measured not merely by the increased extent of the incision, but by the greater facility with which the neck of the bladder will then yield to the pressure of the forceps. If the stone be still too large to be safely extracted it should be broken either by a pair of strong forceps with much projecting teeth, or the instrument contrived by Mr. Earle, and described in the 11th Vol. of the Medico-Chirurgical Transactions.

"An exclusive preference is not to be given to the gorget or to the knife for the incision of the prostate. With either instrument, skilfully used, the operation may be well done. With a gorget, properly constructed, there is no risk of wounding the pudendal artery or the rectum, because the limits of the incision are determined by the dimension and form of the instrument. With a knife, in an inexperienced hand, there is not so much certainty of confining the incision within its proper limits.

"A comparison of the gorget with the knife, so far instituted, is favourable to the former; but to the narrow-bladed and beaked knife, first used by Mr. Blizard, an advantage belongs, which a gorget, from the width of its blade, cannot possess. The knife enters the bladder, as Mr. Blizard was accustomed to remark, as easily as a probe. The gorget, on the other hand, must meet resistance in passing through the prostate. Very much less, however, will this resistance be than it has been usually represented when the gorget has been properly made, and it is guided with skill.

"For the young subject, or for a thin adult, the knife is especially suited. It is also to be preferred for any case in which the bladder is closely contracted upon the stone. But for a very fat, or for an old subject, in whom, by the enlargement of the prostate, or the dilatation of the rectum, the bladder is raised much above its natural situation, the gorget is better adapted, on account of the great distance from the perineum at which the prostate and neck of the bladder are, in such instances, situated.

"Other methods of dividing the prostate, and other instruments for doing it, have been recommended: doubtless, some of these are good; but, for the mention of them here, consistently with my present object, it would be necessary for them to be better than those which have been described, and I have not been able to satisfy myself that they are so." 19.

The third section contains some very judicious remarks upon the sound, staff, gorget, and beaked knife, remarks which we would recommend to the attention of the young surgeon. His success will be found to depend much more on these minutiæ than a person at first would imagine, and often have we seen an able and dexterous operator embarrassed by an ill-adapted beak and groove. We must now close our notice of this highly meritorious and modest production. The extent of our analysis shews the esteem in which we hold it, and we trust we may say without suspicion of adulation, that the present *brochure* of Mr. Stanley does no discredit to the author of the "Manual of Anatomy." We strongly recommend those students who are engaged in the operations on the dead body, to peruse these instructions before they pass to the performance of lithotomy.

